# Epidemiological and Epizootological Monitoring and Spatiotemporal Dynamics of Plague in Natural Foci of Kazakhstan

**DOI:** 10.3390/pathogens15070685

**Published:** 2026-06-29

**Authors:** Ziyat Abdel, Zauresh Zhumadilova, Raikhan Mussagalieva, Aigul Abdirassilova, Svetlana Issaeva, Galina Kovaleva, Bolatbek Baitursyn, Beck Abdeliyev, Temirkhan Sagidulin, Nurbol Shaki, Damira Shonshabayeva, Alim Saduakas, Tatyana Meka-Mechenko

**Affiliations:** 1 M. Aikimbayev’s National Scientific Center for Especially Dangerous Infections, 14 Zhakhanger St., Almaty A35P0K3, Kazakhstan; abdelziyat767@gmail.com (Z.A.); zzbgdirect@nscedi.kz (Z.Z.); raikhansafar@gmail.com (R.M.); aigul.abdirassilova@mail.ru (A.A.); s.isaeva64@mail.ru (S.I.); kgala77@mail.ru (G.K.); b.bola-1993@mail.ru (B.B.); abdelbeck@gmail.com (B.A.); nscedi@nscedi.kz (T.S.); nurbol.shakiy@gmail.com (N.S.); 2 Zhambyl Anti-Plague Station, Taraz 080010, Kazakhstan; damira04.04@mail.ru (D.S.); alim.saduakas@mail.ru (A.S.)

**Keywords:** plague, *Yersinia pestis*, natural foci, epizootology, rodents, fleas, resistance factors

## Abstract

Plague remains a significant natural focal zoonotic infection with continuing epidemiological relevance in the Republic of Kazakhstan. This study provides a comprehensive assessment of epizootological dynamics in natural plague foci during 2020–2025 through the integration of historical epidemiological data, phenotypic and molecular characterization of *Yersinia pestis*, and GIS-based spatial analysis. The study utilized long-term surveillance data (1920–2025), epidemiological records of human plague cases (1926–2003), phenotypic analysis of 1526 strains, and whole-genome sequencing of 75 representative isolates. Epizootological monitoring demonstrated high surveillance coverage and stable monitoring capacity, together with a marked increase in the application of molecular diagnostic methods. By 2025, both the number and isolation rate of *Y. pestis* strains increased substantially, while the epizootically active area expanded in 2024–2025, although the overall long-term trend in active area was not statistically significant. Despite these fluctuations, *Y. pestis* populations remained highly stable, with 94.9% phenotypically typical and 97.5% genotypically typical strains, and no evidence of antimicrobial resistance. Spatial autocorrelation analysis using Moran’s I revealed significant clustering of epizootics (Moran’s I = 0.1627; z = 4.39; *p* < 0.001), indicating non-random spatial distribution and localized zones of increased epizootic activity. No human plague cases have been recorded since 2003 during the period of sustained epidemiological surveillance and control measures. These findings highlight the potential utility of integrating spatial modeling and molecular surveillance into risk-oriented plague monitoring and control strategies.

## 1. Introduction

Plague is a natural focal zoonotic infection caused by *Yersinia pestis* and remains an important public health concern in several regions of the world, including Central Asia, Africa, and the Americas [1,2,3,4,5,6]. Despite major advances in surveillance and molecular diagnostics, the pathogen continues to circulate in wildlife populations and periodically causes human outbreaks.

Kazakhstan contains one of the largest plague-endemic territories worldwide. Natural plague foci occupy approximately 1.1 million km^2^, representing nearly 40% of the country’s area and more than half of the total plague-endemic territory within the Commonwealth of Independent States [7,8,9]. These foci include steppe, desert, semi-desert, and high-mountain ecosystems that provide favorable conditions for the long-term persistence and circulation of *Y. pestis* [10,11]. Several foci are transboundary and connected with neighboring endemic areas in Kyrgyzstan, Mongolia, China, and Russia [12,13,14]. Although the last human plague case in Kazakhstan was reported in 2003 [7], epizootics among wild rodents and their fleas continue to be detected annually [10,15,16].

Previous studies in Kazakhstan have described the distribution of natural plague foci, historical patterns of human cases, ecological characteristics of reservoir hosts, and genomic diversity of *Y. pestis* [17,18,19,20]. Recent advances in geographic information systems (GIS), spatial statistics, and whole-genome sequencing (WGS) have created new opportunities to integrate ecological, epidemiological, and molecular data for more comprehensive assessment of plague dynamics and risk [21,22,23,24,25]. These approaches are complemented by standardized epidemiological surveillance systems and international guidelines for plague monitoring and control, which provide the methodological framework for risk assessment and public health decision-making [26,27,28].

Despite the availability of extensive long-term epidemiological and epizootological surveillance data in Kazakhstan, these information sources have rarely been analyzed together with contemporary genomic and spatial approaches within a unified analytical framework. Integrating historical records of human cases, current epizootological monitoring, whole-genome sequencing, and GIS-based spatial analysis can provide a more complete understanding of pathogen circulation and improve risk-oriented surveillance and prevention.

The aim of this study was to assess the current epizootological situation in the natural plague foci of Kazakhstan during 2020–2025, to analyze long-term epidemiological and epizootological trends, and to integrate molecular and spatial data in order to characterize pathogen dynamics and support evidence-based public health decision-making.

## 2. Materials and Methods

### 2.1. Epizootological Monitoring and Field Sampling

**Figure 1:**
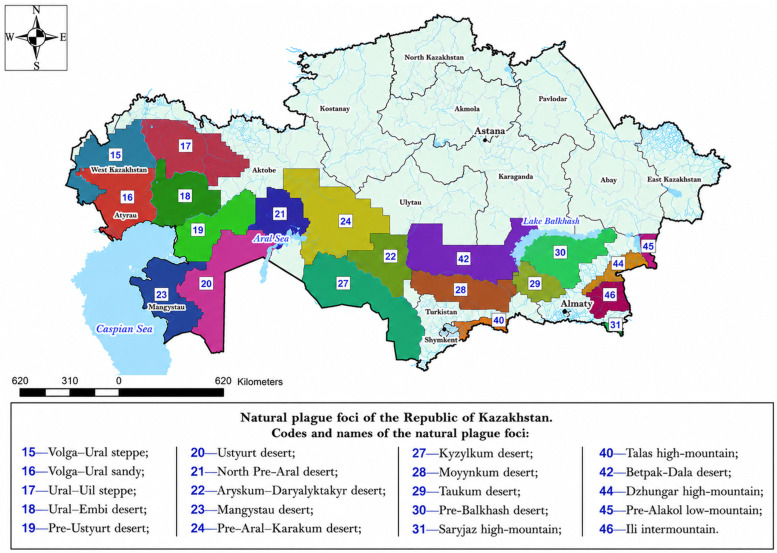
Natural plague foci of the Republic of Kazakhstan.

The study was conducted within the natural plague foci of Kazakhstan (Figure 1), where epizootological monitoring was performed using standard field, laboratory, and molecular methods, including rodent trapping, ectoparasite collection, bacteriological, serological, and real-time PCR analyses.

The experimental and analytical base of the research was the Central Reference Laboratory of the M. Aikimbayev National Scientific Center for Especially Dangerous Infections under the Ministry of Health of the Republic of Kazakhstan.

The study is based on extensive long-term observations derived from epidemiological monitoring of human plague cases (1926–2003) and epizootological surveillance (1920–2025) within the natural plague foci of Kazakhstan. These include 6 major natural foci and 15 autonomous foci, within which more than 100 landscape-epizootological regions have been delineated.

### 2.2. Study Area and Characteristics of Natural Plague Foci

The study area encompasses the principal natural plague foci of the Republic of Kazakhstan, represented by steppe, desert, and high-mountain ecosystems. The epizootological structure of these foci is characterized by a diversity of primary hosts (rodents and marmots), as well as the circulation of different biovars and phylogenetic lineages of *Y. pestis*.

The main characteristics of the natural foci, including focus types, geographic distribution, primary hosts, biochemical properties, and phylogenetic affiliation of the pathogen, are presented in Table 1.

### 2.3. Characterization of Isolated Strains

Phenotypic characterization was performed on a total of 1526 *Y. pestis* strains isolated from natural plague foci in Kazakhstan between 2010 and 2025. The analyses included assessment of cultural, morphological, biochemical, virulence, and plasmid-associated characteristics in accordance with standard methodologies [29,30].

Genomic DNA was extracted using the QIAamp DNA Mini Kit (Qiagen, Germantown, MD, USA). DNA libraries were prepared using the Nextera XT DNA Library Preparation Kit (catalog No. FC-131-1024) according to the manufacturer’s protocol. Sequencing was performed on the Illumina MiSeq platform using the MiSeq Reagent Kit v3 (600 cycles; catalog No. MS-102-3003).

Raw sequencing reads were quality-checked and trimmed using Trimmomatic v0.36. High-quality reads were subjected to de novo assembly using SPAdes v3.15.0 and reference-based mapping using Bowtie2 v2.4.1. The genome of *Y. pestis* strain SCPM-O-B-6899 (GenBank accession CP045145) was used as the reference sequence for read mapping and consensus sequence generation. Consensus assemblies and variant calling were performed using BCFtools v1.18.

SNPs and polymorphic loci were filtered according to the following quality criteria: QUAL ≥ 30, DP ≥ 10, MQ ≥ 40, and MQ0F ≤ 0.1. Genome assemblies were screened for major virulence determinants, including the caf1 gene and plasmid-associated markers, as well as genes associated with antimicrobial resistance. Quality control included evaluation of sequence coverage, read depth, and contamination. Only genomes meeting internal quality criteria were included in the downstream analysis.

The resulting genomic data were used for lineage assignment, comparative analysis of core genomic variation, and screening for virulence and antimicrobial resistance determinants. Detailed descriptions of the sequencing procedures, assembly pipelines, and genomic analyses of *Y. pestis* isolates from Kazakhstan natural plague foci were published previously [10,18,19].

### 2.4. Quality Control of the Study

Quality control was ensured through the use of reference and control strains maintained in the live culture collection of the M. Aikimbayev National Scientific Center for Especially Dangerous Infections. These included reference strains of *Y. pestis* from different autonomous foci of Kazakhstan, the vaccine strain *Y. pestis* EV, and *Y. pseudotuberculosis*.

In addition, reference sequence fragments from four well-characterized strains representing the principal biovars of the plague pathogen were used: Pestoides F strain (Microtus/Antiqua biovar), Nepal516 strain (Antiqua biovar), KIM10 strain (Medievalis biovar), and CO92 strain (Orientalis biovar).

### 2.5. Epidemic Year Index (EYI)

The Epidemic Year Index (EYI) was used as a descriptive comparative indicator to characterize the long-term frequency of human plague occurrence in different regions and natural foci. EYI was calculated as the proportion of years with at least one recorded human plague case during a defined observation period:

$$EYI=\frac{N\;epidemic\;years}{N\;total\;years}$$

where N epidemic years is the number of years with recorded human plague cases and N total years is the total number of years under observation.

EYI was used to facilitate relative comparisons among regions and time periods. It is not a validated predictive risk index and does not account for case counts, population at risk, surveillance effort, geographic extent, or temporal changes in reporting practices. Accordingly, EYI was interpreted as a descriptive indicator and was used as one component of the broader epidemiological risk assessment.

### 2.6. Risk Assessment

Risk assessment was performed using a comprehensive approach integrating epizootological, epidemiological, and spatial–analytical indicators. The analysis incorporated the intensity of epizootic activity, frequency of pathogen detection, distribution of host and vector populations, and historical epidemiological data.

A risk-oriented framework was applied to classify natural plague foci according to relative levels of epidemiological activity, taking into account environmental, biological, and socio-demographic factors. Spatial assessment was conducted using GIS-based methods, enabling the identification of areas with elevated epizootic activity and the evaluation of spatial distribution patterns. Spatial analysis and cartographic visualization were performed using ArcGIS 10.8 (Esri, Redlands, CA, USA).

### 2.7. Statistical Analysis

Statistical analyses were performed using R version 4.3.2 (R Foundation for Statistical Computing, Vienna, Austria) with the packages *stats*, *MASS*, *ggplot2*, and *dplyr*. Because most variables did not follow a normal distribution, non-parametric statistical methods were applied.

Comparisons between independent groups were conducted using the Mann–Whitney U test for two-group comparisons and the Kruskal–Wallis test for comparisons involving more than two groups. Associations between continuous variables were assessed using Spearman’s rank correlation analysis.

Temporal trends and relationships between surveillance indicators and epizootic activity were analyzed using Poisson regression models. The dependent variables included the number of plague detections and the epizootically active area, whereas independent variables included year, surveillance intensity, and the Epidemic Year Index (EYI). To account for differences in surveillance effort between regions and study periods, logarithmic offset terms based on the number of samples examined and surveyed area were incorporated into the models where appropriate.

Regression results were reported as regression coefficients (β) and incidence rate ratios (IRRs) with corresponding 95% confidence intervals (95% CI) and *p*-values. A *p*-value < 0.05 was considered statistically significant.

## 3. Results

### 3.1. Epizootological Monitoring

Epizootological monitoring of natural plague foci in the Republic of Kazakhstan during 2020–2025 was characterized by broad territorial coverage, stable surveillance activities, and sustained implementation of preventive measures (Table 2).

Epizootic area was defined as the area in which active circulation of *Y. pestis* was confirmed by bacteriological, serological, or molecular methods during annual surveillance.

The total surveyed area ranged from 834.6 to 896.2 thousand km^2^ and remained relatively stable throughout the study period. The area of epizootically active territories varied from 3.0 to 8.9 thousand km^2^, corresponding to 0.34–0.99% of the surveyed area. Minimum values were observed in 2022, with higher values recorded in 2024–2025. However, the overall trend in epizootically active area over 2020–2025 was not statistically significant and was therefore interpreted cautiously.

Preventive measures were implemented systematically throughout the study period. Between 116 and 196 protective zones were established annually, covering 344.4–595.0 km^2^. More than 500 settlements were surveyed each year, and the volumes of rodent and flea infestation surveys, deratization, and disinsection remained consistently high. Vaccination of humans and camels was performed annually, with a marked increase in camel vaccination in 2025.

Field investigations and laboratory diagnostics are summarized in Table 3. The volumes of zoological and parasitological investigations remained substantial: up to 156.9 thousand rodents and more than 1.5 million ectoparasites were examined annually. Over the study period, the number of rodents examined gradually decreased, whereas the volume of ectoparasite investigations remained consistently high.

Laboratory diagnostics showed a moderate increase in bacteriological testing, reaching 231.1 thousand samples in 2025. The number of isolated *Y. pestis* strains increased from 31 in 2020 to 91 in 2025, and the isolation rate rose from 16.6 to 39.4 isolates per 10^5^ bacteriologically examined samples. The use of molecular methods expanded considerably, with the number of real-time PCR assays increasing from 1.2 thousand in 2020 to 53.4 thousand in 2025. Real-time PCR positivity ranged from 0.6 to 11.7 positive results per 10^3^ samples.

Serological monitoring showed pronounced interannual variability, with the number of positive serological results ranging from 51 in 2022 to 240 in 2020 and increasing again to 212 in 2025.

Increased detection of *Y. pestis* was reflected in the rise in the number of isolated strains from 31 in 2020 to 91 in 2025, together with an increase in real-time PCR results from 14 to 71. The highest values were recorded in 2025.

**Figure 2:**
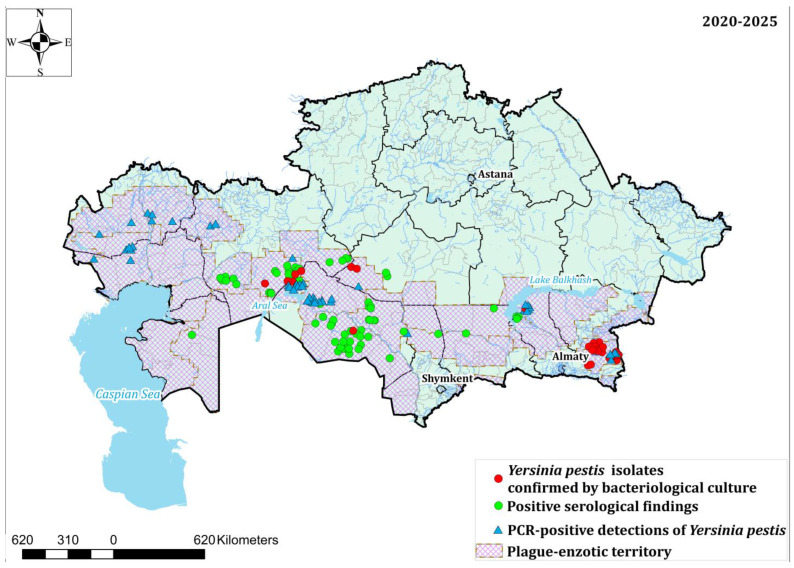
Results of epizootological monitoring: detection of *Yersinia pestis* strains, seropositive animals (F1 fraction), and plague antigen.

Spatial analysis revealed pronounced focal heterogeneity (Figure 2). During 2020–2022, epizootic activity was localized, primarily confined to the Ili intermontane, Kyzylkum, and Arys-Kum–Daryalyktakyr foci. In contrast, during 2023–2025, broader geographic distribution of pathogen circulation was observed, involving the North Aral, Balkhash, and Aral–Karakum foci.

By 2025, the North Aral focus became dominant, accounting for more than 75% of all isolated strains and representing the principal center of epizootic activity.

Serological monitoring revealed pronounced interannual variability: the number of seropositive animals decreased to 51 in 2022 but subsequently increased to 212 in 2025, with the highest values observed in the North Aral and Moyynkum foci. PCR-based diagnostics likewise demonstrated broader pathogen detection across multiple foci, particularly in 2025.

Overall, the findings indicate the stable functioning of the epizootological monitoring system and increased detection of *Y. pestis* in recent years. This was reflected in higher numbers and isolation rates of *Y. pestis* strains, together with the expanded use of molecular diagnostic methods. Although larger epizootically active areas were recorded in 2024–2025, changes in this indicator were interpreted cautiously because the overall trend over 2020–2025 was not statistically significant.

The observed changes in key epizootological indicators suggest that years with larger epizootically active areas were generally accompanied by higher numbers of isolated *Y. pestis* strains and real-time PCR detections. These patterns should be interpreted in the context of increased surveillance effort and the substantial expansion of molecular testing.

### 3.2. Results of Phenotypic and Molecular-Genetic Studies

During the period 2010–2025, phenotypic characterization was performed on 1526 *Y. pestis* strains isolated from natural plague foci in Kazakhstan, and whole-genome sequencing was conducted on a representative subset of 75 isolates selected to reflect the geographic, temporal, ecological, and phenotypic diversity of the collection (Table 4).

The analysis demonstrated that all studied strains conformed to the typical cultural, morphological, biochemical, and enzymatic characteristics of populations circulating within the Central Asian desert plague focus. The strains fermented glycerol, glucose, mannitol, maltose, and arabinose, did not ferment rhamnose, were pesticinogenic, and lacked denitrification activity.

The majority of strains produced the capsular F1 antigen and exhibited high virulence in laboratory animals (LD_50_ for guinea pigs: 1.0 × 10^6^ CFU; for white mice: 1.6–9.0 × 10^4^ CFU). All strains remained susceptible to antibacterial agents; no antibiotic-resistant or bacteriophage-resistant variants were detected.

At the same time, based on the combined phenotypic characteristics, 94.9% of strains were classified as typical, whereas 5.1% exhibited altered properties. Among the 75 whole-genome sequenced isolates, 97.5% were classified as genotypically typical, whereas 2.5% lacked the caf1 gene (plasmid pFra), which encodes the F1 capsular antigen.

Serological testing for the F1 antigen (indirect hemagglutination assay) showed that 98.4% of strains contained the F1 antigen, with concentrations ranging from 1:1250 to 1:78,125 CFU/mL. The absence of F1 was observed in a limited number of strains, primarily from the Balkhash autonomous focus, as well as in isolated cases from the Ili intermontane, Taukum, and Moyynkum foci.

Strain populations demonstrated a high degree of homogeneity with respect to calcium dependence and pigmentation: the proportion of Ca^2+^-dependent forms was 98.6 ± 2.4%, and pigment-absorbing strains accounted for 94.3 ± 1.7%. The proportion of Pgm^+^ cells was 94.7%, while Pgm^−^ variants constituted 5.3%. Analysis of pesticin production showed that 99.6% of strains produced pesticin I and remained resistant to it, whereas only 0.4% of strains (from the Balkhash focus) did not produce this factor.

Analysis of growth factor requirements revealed that the vast majority of strains (n = 1489) required phenylalanine, methionine, and cysteine. A small number of isolates exhibited dependence on threonine (n = 8) or arginine (n = 29); however, the distribution of these traits did not correlate with the level of epizootic or epidemic activity in the foci.

Comparative analysis showed that in autonomous foci with high epizootic activity and frequent epidemic complications, the frequency of atypical strains was 8.2-fold higher than in foci with low activity.

Overall, the analysis indicates high phenotypic stability across the full collection of 1526 strains and high genotypic stability within the representative subset of 75 whole-genome sequenced isolates.

Atypical strains were more frequently observed in foci with elevated epizootic activity. This association should be interpreted cautiously, as genotypic conclusions are based on a representative rather than statistically random subset of sequenced isolates.

Whole-genome sequencing analysis confirmed the genetic diversity of the investigated *Y. pestis* isolates and supported their differentiation according to geographical origin and epidemiological characteristics. Comparative genomic analysis identified polymorphic loci and phylogenetic clustering patterns consistent with previously described Central Asian plague populations.

Detailed phenotypic, molecular, antimicrobial resistance, and comparative genomic characteristics of these isolates were described previously in dedicated studies [10,18,19].

### 3.3. Epidemiological Analysis

A retrospective analysis of long-term observations derived from epidemiological monitoring of human plague cases (1926–2003) and epizootological surveillance within the natural plague foci of Kazakhstan demonstrated substantial spatial and temporal heterogeneity of the epidemic process. During 1926–2003, more than 560 human plague cases were recorded in the desert plague foci of Kazakhstan, grouped into 82 epidemic outbreaks.

The incidence distribution was markedly uneven across regions. The largest proportion of cases occurred in the Kyzylorda (40.53%) and Almaty (32.22%) regions, whereas substantially lower proportions were observed in the Mangystau (21.24%), Atyrau (4.42%), and Aktobe (1.59%) regions. These regional differences likely reflect ecological variation among natural plague foci, including differences in dominant reservoir hosts, flea vectors, habitat structure, and environmental conditions influencing the persistence and intensity of *Y. pestis* circulation [7,8,9,17,18,19,20].

Analysis of temporal dynamics allowed identification of three major epidemiological stages. The pre-antibiotic period (1926–1948) was characterized by high incidence and large outbreaks (1926, 1929, 1945, 1947, and 1948), accounting for up to 80.7% of all recorded cases, including episodes of human-to-human transmission. The period of stabilized epidemiological surveillance (1950–1990) was associated with a substantial decline in incidence following the introduction of antibiotic therapy and systematic anti-plague measures. During the subsequent period of socio-economic transition (1990–2003), an increase in the proportion of epidemic years was observed, potentially associated with reduced effectiveness of epidemiological control and deterioration of sanitary and hygienic conditions. Overall, a statistically significant reduction in incidence following the implementation of antibiotic therapy and structured epidemiological surveillance was identified (*p* < 0.05), followed by fluctuations during the post-Soviet period.

To provide a comparative assessment of long-term epidemiological activity, the Epidemic Year Index (EYI) was calculated as the proportion of years with recorded human plague cases relative to the total observation period. During 1926–2003, the mean EYI was approximately 49%, indicating substantial long-term variability in epidemic activity within natural plague foci.

**Figure 3:**
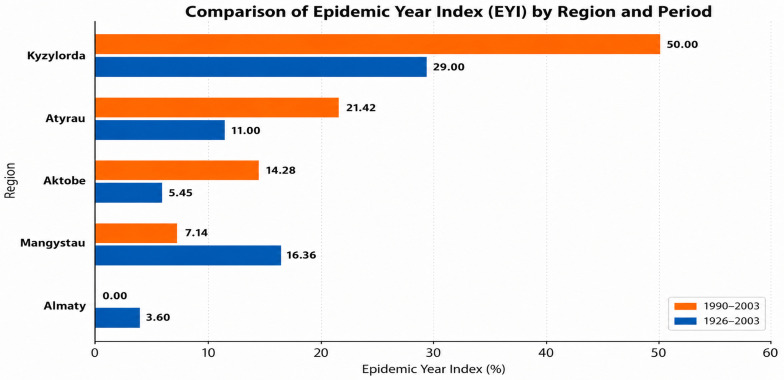
Regional comparison of the Epidemic Year Index (EYI) in Kazakhstan across two periods (1926–2003 and 1990–2003). Horizontal bars represent the proportion of epidemic years across regions.

Regional analysis demonstrated pronounced heterogeneity in EYI values (Figure 3): Kyzylorda region—29.0%, Atyrau—11.0%, Aktobe—5.45%, Mangystau—16.36%, and Almaty—3.6%. During the post-Soviet period (1990–2003), a redistribution of epidemiological activity was observed: Kyzylorda—50.0%, Atyrau—21.42%, Aktobe—14.28%, Mangystau—7.14%, and Almaty—0%. For comparative purposes, the post-Soviet period (1990–2003) was analyzed separately because of major socio-economic and public health system changes that potentially influenced plague surveillance and epidemiological dynamics.

Comparison of the two periods demonstrated increased epidemiological activity in the Kyzylorda, Atyrau, and Aktobe regions after 1990, together with decreased activity in the Mangystau and Almaty regions. These regional and temporal differences were statistically significant (*p* < 0.05). The EYI served as a descriptive comparative indicator of long-term epidemiological activity and regional heterogeneity.

Human cases were strictly associated with periods of increased epizootic activity in natural plague foci. All recorded cases occurred exclusively within zones of active pathogen circulation, confirming the secondary (spillover) nature of the epidemic process and its dependence on epizootic dynamics rather than sustained transmission within the human population.

The observed patterns have a clear ecological basis and are consistent with the natural focal nature of plague [2,7,8,9,17,18,19,20]. They are driven by fluctuations in the population dynamics of primary reservoirs (particularly Rhombomys opimus), the activity of vector populations (fleas), and the climatic and landscape characteristics of arid ecosystems [10,11,18,19]. Cyclical changes in rodent abundance lead to periodic activation of epizootics, which in turn determines the probability of human infections [1,2,21,22].

Socio-economic factors also exert an additional influence. The 1990s were characterized by reduced effectiveness of epidemiological surveillance, deterioration of sanitary and hygienic conditions, and increased human contact with natural foci of infection, resulting in a higher proportion of epidemic years and increased epidemiological risk (*p* < 0.05) [7,8,9,17,18,19,20].

Despite the continued epizootic activity in natural foci, the last human case of plague in the Republic of Kazakhstan was recorded in 2003. This indicates the high effectiveness of the national epidemiological surveillance system and the successful implementation of an integrated One Health approach.

In summary, the epidemic process of plague in Kazakhstan is characterized by pronounced spatiotemporal heterogeneity and is determined by the structure of natural foci. Following the introduction of antibiotic therapy and systematic epidemiological surveillance, a statistically significant reduction in incidence was observed (*p* < 0.05). The Epidemic Year Index (EYI) serves as a descriptive comparative indicator of long-term epidemiological activity and regional heterogeneity. Human incidence exhibits a secondary (spillover) pattern and is strictly linked to phases of epizootic activity. The dynamics of the epidemic process are governed by a combination of ecological and socio-economic factors.

It is important to emphasize that, despite the persistent epizootic circulation of plague in natural foci among wild animals, no human cases have been recorded in Kazakhstan since 2003. This reflects the effectiveness of state-level organization of surveillance and control measures, as well as the implementation of a comprehensive, integrated system of anti-plague interventions carried out by the national anti-plague service.

### 3.4. Assessment of Epidemiological Risk and Epizootic Activity

A retrospective assessment of epizootic and epidemic manifestations of plague in the natural foci of Kazakhstan (total area approximately 1.1 million km^2^) allowed their differentiation according to levels of epidemiological risk. The classification was based on the frequency of epizootic events and the intensity of human plague cases.

Based on long-term data analysis, all autonomous plague foci were categorized into five risk levels: very high, high, medium, low, and very low. The highest risk level was characteristic of foci with repeated epizootic activity across all epizootic cycles and a high number of human cases, whereas the very low risk level corresponded to areas with sporadic epizootic manifestations and no recorded human infections.

For spatial and quantitative risk assessment, a total of 9327 sectors within natural plague foci were analyzed. Sector classification was performed based on three key parameters: (1) presence of epizootic activity, (2) occurrence of human cases, and (3) population density. Based on the combined evaluation of these parameters, the level of potential epidemic hazard (PEH) was determined.

**Figure 4:**
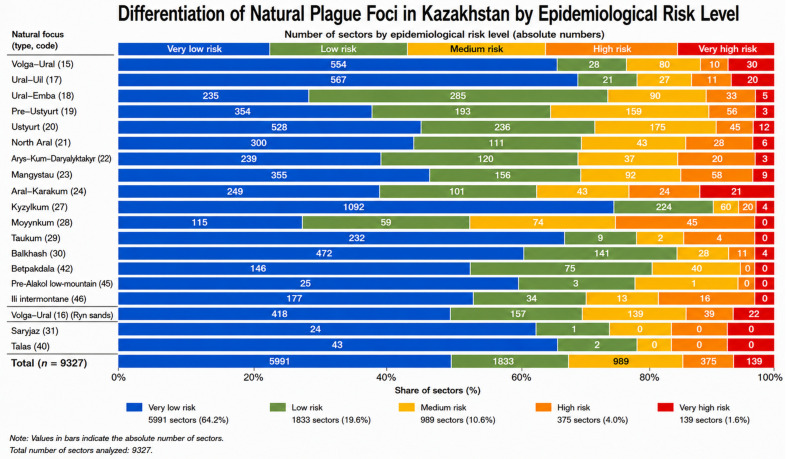
Differentiation of natural plague foci in Kazakhstan by epidemiological risk level.

To support risk-oriented planning of surveillance and preventive measures, all 9327 sectors within the natural plague foci of Kazakhstan were classified into five epidemiological risk categories based on the combined assessment of long-term epizootic activity, historical human plague cases, and population density. Figure 4 presents the distribution of sectors across these categories for each natural plague focus. Numbers within the bars indicate the absolute number of sectors in each category.

According to the obtained results, 139 sectors were classified as very high risk, 375 as high risk, 989 as medium risk, 1833 as low risk, and 5991 as very low risk. The analysis demonstrated pronounced heterogeneity in the epidemiological significance of natural plague foci. Very high-risk sectors were concentrated primarily in the Volga–Ural steppe, Volga–Ural sandy, Ural–Uil steppe, and North Aral foci, reflecting the long-term persistence of epizootic activity and documented human plague cases. In contrast, high-mountain foci such as Saryjaz and Talas were characterized almost exclusively by very low-risk sectors. Among sandy desert foci, Kyzylkum contained the largest number of sectors (n = 1400), the majority of which were classified as very low risk, although medium- and high-risk sectors were also present. These results indicate that most sectors remain at low epidemiological risk, while a limited number of geographically concentrated sectors account for the highest potential for epidemic complications.

**Figure 5:**
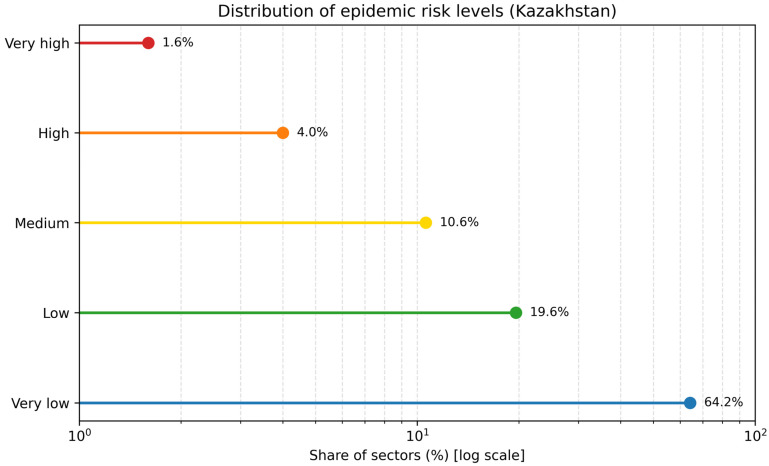
Percentage distribution of 9327 sectors in natural plague foci of Kazakhstan by epidemiological risk level. A lollipop chart with a logarithmic scale illustrates the proportion of sectors across five risk categories. Color coding reflects increasing epidemiological risk from very low (blue) to very high (red). Due to the highly skewed distribution of sector proportions, a logarithmic scale was applied to improve visualization.

The distribution of sectors across risk categories (Figure 5) showed that the majority of territories fall within the very low risk category (64.2%), whereas areas with high and very high risk account for only 5.6%. Despite their relatively small proportion, these areas with elevated epidemiological activity represent the greatest epidemiological significance as potential sources of outbreak emergence.

Analysis of spatial distribution revealed that approximately 28% of the territory of natural plague foci is characterized by epizootic activity, while epidemic manifestations are recorded in only 3.7% of the area. This finding confirms that human plague cases represent a localized phenomenon, occurring only under specific combinations of epizootic, environmental, and socio-demographic factors.

Further analysis indicated that foci classified as high and very high risk are characterized by persistent and recurrent epizootic activity, whereas low- and very low-risk foci exhibit sporadic or irregular epizootic patterns. This highlights the pronounced spatial heterogeneity of epidemiological risk across natural plague foci.

Overall, epizootological differentiation and epidemiological zoning of plague foci represent essential tools for planning and optimizing preventive (anti-plague) measures, enabling the identification of priority areas for surveillance and intervention. Epidemiological zoning enables the identification of priority areas for surveillance by integrating epizootic dynamics with demographic and environmental risk factors.

### 3.5. Spatial Analysis of Epizootic Activity Using GIS Technologies

A comprehensive spatial analysis of the epizootic activity of *Y. pestis* in the natural foci of Kazakhstan during 2020–2025 was performed using geoinformation and spatial statistical methods in ArcGIS 10.8 (Esri, Redlands, CA, USA), enabling the identification of pathogen circulation patterns and spatial distribution characteristics of epizootic activity.

**Figure 6:**
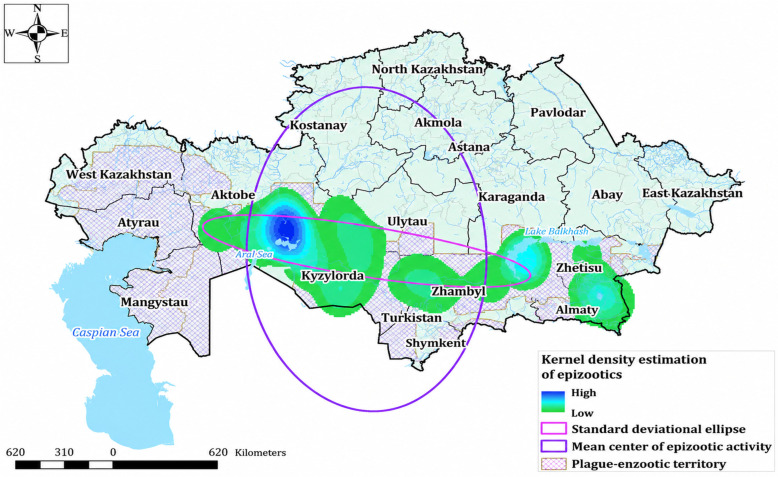
Frequency of epizootic occurrence within one standard deviation from the mean center and the directional distribution ellipse of epizootics (2020–2025), Mercator coordinate system.

Application of the standard distance method allowed for the assessment of spatial dispersion and the central tendency of the distribution of epizootic occurrence points. As shown in Figure 6, the majority of cases were concentrated within the area corresponding to one standard deviation from the mean center, indicating the presence of stable cores of epizootic activity. The identification of such zones is of critical importance for defining priority areas for epizootological surveillance.

To assess the directionality of epizootic spread, the directional distribution method was applied. The resulting ellipse (Figure 6) demonstrates a pronounced spatial orientation of epizootics from the southeast to the northwest, indicating the presence of a persistent vector of infection spread. This directional pattern may be driven by a combination of natural factors, including landscape features, migration dynamics of primary reservoirs (particularly *Rhombomys opimus*), as well as the influence of anthropogenic processes.

Spatial autocorrelation analysis using the global Moran’s I statistic revealed statistically significant clustering of epizootics (Moran’s I = 0.1627; z = 4.39; *p* < 0.001), confirming the non-random nature of their spatial distribution and the presence of geographically localized zones of increased epizootic activity. The identified spatial structure highlights the need to prioritize surveillance and preventive interventions within these clusters.

**Figure 7:**
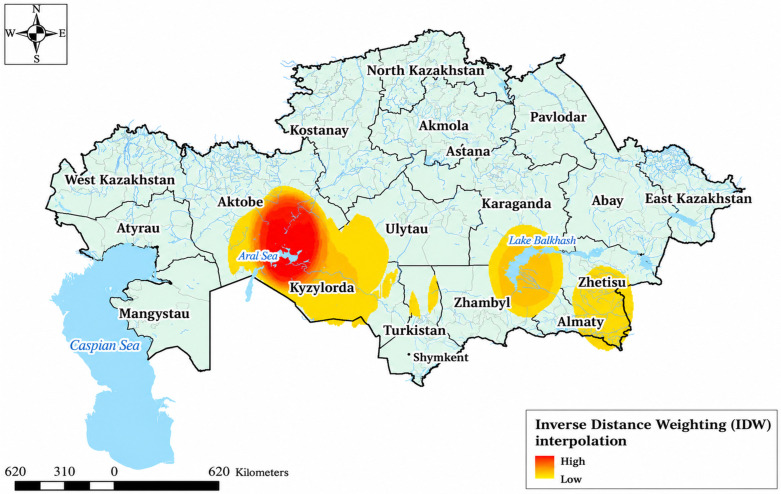
Interpolated spatial distribution of epizootic activity based on the inverse distance weighting (IDW) method, Mercator coordinate system.

To assess the spatial distribution of epizootic activity, the inverse distance weighting (IDW) method was employed. The resulting interpolated spatial distribution map (Figure 7) illustrates variation in epizootic activity across the study area.

The modeling results indicated that the highest levels of epizootic activity were associated with the North Aral, Betpakdala, Moyynkum, and Kyzylkum autonomous foci. At the same time, elevated epizootic activity was observed in adjacent territories, including the Volga–Ural sandy, Pre-Ustyurt, and Ustyurt foci.

Importantly, the areas with elevated epizootic activity identified through interpolation spatially correspond to the directional distribution of epizootics, confirming the internal consistency of the applied analytical approaches. Despite certain limitations related to the uneven distribution of input data, the applied methodology adequately captures the key patterns of pathogen circulation.

Thus, the use of GIS technologies and spatial statistical methods enabled the identification of stable epizootic cores, the determination of the directionality of infection spread, and the development of a spatial assessment model of epizootic activity. The obtained results provide a scientific basis for optimizing epizootological surveillance, improving resource allocation, and enhancing the planning of preventive measures in natural plague foci.

## 4. Discussion

The present study integrates long-term epidemiological, epizootological, molecular, and spatial data, providing a comprehensive understanding of plague persistence in one of the largest natural plague foci globally. The findings highlight a notable epidemiological pattern: increasing epizootic activity without human cases since 2003, coinciding with sustained surveillance systems and targeted anti-plague interventions. Similar patterns have been reported in other endemic regions where intensive surveillance and control activities reduce the likelihood of zoonotic spillover [2,31].

The observed increase in epizootic activity during 2020–2025, including expansion of affected territories and increased pathogen detection, aligns with known ecological cycles of rodent populations and flea vectors in arid ecosystems [32,33]. In particular, the intensification of molecular diagnostic approaches contributed to improved detection sensitivity, consistent with global trends in zoonotic disease surveillance [34].

Spatial modeling identified areas with persistently elevated epizootic activity, particularly within the North Aral, Volga–Ural sandy, Pre-Ustyurt, and Ustyurt autonomous plague foci. Elevated activity was also observed in the Betpakdala, Moyynkum, and Arys-Kum–Daryalyktakyr foci. These spatial patterns were consistent with the identified clustering and directional distribution of epizootics.

Previous studies have suggested that climatic and environmental factors may influence the distribution and dynamics of plague reservoirs and vectors in endemic regions [35,36]. These observations highlight the importance of considering environmental variability within long-term surveillance frameworks.

The high phenotypic and genotypic stability of *Y. pestis* populations observed in this study is consistent with previous genomic investigations demonstrating limited evolutionary divergence in natural plague foci [37,38]. At the same time, the increased frequency of atypical strains in highly active foci may reflect localized ecological pressures and ongoing microevolutionary processes [39].

Spatial analysis demonstrated significant clustering and structured spatial organization of epizootic activity, supporting the presence of persistent localized centers of pathogen circulation. A consistent southeast–northwest directional spread was also identified, indicating non-random dynamics of epizootic processes. Comparable spatial patterns have been described in Central Asia and China, where landscape connectivity and reservoir migration play important roles in pathogen distribution [40,41].

The epidemiological analysis confirms the classical natural-focal character of plague circulation, with human cases closely associated with epizootic activity. The Epidemic Year Index (EYI) served as a useful descriptive indicator for comparative assessment of long-term epidemiological activity and regional heterogeneity, consistent with approaches applied in other zoonotic systems [42]. The increase in epidemiological activity during the post-Soviet period additionally highlights the potential influence of socio-economic factors on plague dynamics [43].

Overall, these findings support the integration of ecological, molecular, and spatial approaches within a One Health framework. The combination of traditional surveillance, molecular diagnostics, and GIS-based spatial analysis represents an important tool for risk-oriented plague monitoring and proactive disease control, particularly under conditions of environmental and climatic change.

## 5. Conclusions

Plague in Kazakhstan remains a persistent natural focal zoonotic infection characterized by stable circulation in wildlife populations and pronounced spatial heterogeneity.

Despite the observed intensification of epizootic activity in recent years, including expansion of affected areas and increased pathogen detection, no human cases have been recorded since 2003. This demonstrates the high effectiveness of the national epizootological and epidemiological surveillance system and the successful implementation of comprehensive anti-plague measures.

The study confirms the high phenotypic and genotypic stability of *Y. pestis* populations, with a low proportion of atypical variants and no evidence of antimicrobial resistance, supporting a conserved evolutionary pattern of the pathogen.

Spatial analysis identified stable epizootic cores, significant clustering, and a consistent directional spread of infection, while spatial modeling identified zones with persistently elevated epizootic activity, primarily in western desert regions.

The Epidemic Year Index (EYI) provided a descriptive comparative measure of long-term epidemiological activity, highlighting regional heterogeneity and the association between human cases and epizootic dynamics.

Overall, the integration of long-term monitoring data, molecular diagnostics, and GIS-based spatial analysis provides a robust scientific framework for risk-oriented surveillance and proactive plague control. Continued development of these approaches within a One Health framework is essential to prevent the re-emergence of human plague in endemic regions.

## Figures and Tables

**Table 1:** Epizootological characteristics of natural plague foci in Kazakhstan: focus types, primary hosts, biovars, and genetic lineages of *Yersinia pestis*.

Natural Focus (Type, Code)	Location of Enzootic Territory/Region	Primary Host	Subspecies, Biovar, Phylogenetic Lineage	Biochemical Characteristics
Steppe foci: Volga–Ural (15), Ural–Uil (17)	West Kazakhstan, Aktobe regions	Little ground squirrel (*Spermophilus pygmaeus*)	Main subspecies, Medievalis biovar, 2.MED1	Do not ferment rhamnose; do not reduce nitrates; ferment arabinose and glycerol. Virulent, epidemiologically significant
Sandy desert foci: Ural–Emba (18), Pre-Ustyurt (19), Ustyurt (20), North Aral (21), Arys-Kum–Daryalyktakyr (22), Mangystau (23), Aral–Karakum (24), Kyzylkum (27), Moyynkum (28), Taukum (29), Balkhash (30), Betpakdala (42), Pre-Alakol low-mountain (45), Ili intermontane (46)	Atyrau, Aktobe, Almaty, East Kazakhstan, Zhetysu, Zhambyl, Karaganda, Kyzylorda, Mangystau, Turkestan, Ulytau regions	Great gerbil (*Rhombomys opimus*)	Main subspecies, Medievalis biovar, 2.MED1	Do not ferment rhamnose; do not reduce nitrates; ferment arabinose and glycerol. Virulent, epidemiologically significant
Sandy desert (Ryn sands): Volga–Ural (16)	West Kazakhstan, Atyrau regions	Midday gerbil (*Meriones meridianus*), tamarisk gerbil (*M. tamariscinus*), red-tailed gerbil (*M. erythrourus*)	Main subspecies, Medievalis biovar, 2.MED1	Do not ferment rhamnose; do not reduce nitrates; ferment arabinose and glycerol. Virulent, epidemiologically significant
High-mountain focus: Saryjaz (31)	Almaty region	Gray marmot (*Marmota baibacina*)	Main subspecies, Antiqua biovar, 0.ANT5	Do not ferment rhamnose; reduce nitrates; ferment arabinose and glycerol. Virulent, epidemiologically significant
High-mountain focus: Talas (40)	Zhambyl, Turkestan regions	Red marmot (*Marmota caudata*)	Main subspecies, Medievalis biovar, 2.MED1	Do not ferment rhamnose; do not reduce nitrates; ferment arabinose and glycerol. Virulent, epidemiologically significant
	Zhambyl, Turkestan regions	Silver vole (*Alticola argentatus*)	Central Asian subspecies, Talas biovar (Pestoides), 0.PE4t	Ferment rhamnose; do not reduce nitrates; do not ferment arabinose; ferment glycerol. Virulent, epidemiologically significant

**Table 2:** Operational indicators of epizootological surveillance and preventive measures in natural plague foci of Kazakhstan, 2020–2025.

Indicator	Unit	2020	2021	2022	2023	2024	2025
Total surveyed area	10^3^ km^2^	834.60	848.49	888.48	845.59	889.48	896.18
Epizootic area	10^3^ km^2^	7.1	5.4	3.0	3.8	5.7	8.9
Epizootic area	% of surveyed area	0.85	0.64	0.34	0.45	0.64	0.99
Protective zones established	n	152	116	196	154	177	180
Total area of protective zones	km^2^	344.4	354.1	595.0	546.4	394.0	423.2
Settlements surveyed	n	505	507	554	535	521	550
Rodent infestation surveys	10^3^ m^2^	5710.1	4510.0	4560.0	4530.0	4510.0	4544.0
Flea infestation surveys	10^3^ m^2^	4502.5	3265.0	3268.0	3265.0	3288.0	3272.0
Settlement deratization	10^3^ m^2^	1605.0	1745.0	1755.0	1745.0	1769.0	1761.39
Settlement disinsection	10^3^ m^2^	745.0	805.1	899.7	898.5	899.8	904.49
Humans vaccinated	n	94,032	82,331	60,946	61,802	83,889	64,968
Camels vaccinated	n	87,875	91,547	89,546	90,268	89,864	155,059

**Table 3:** Field investigation and laboratory indicators of *Y. pestis* detection in natural plague foci of Kazakhstan, 2020–2025.

Indicator	Unit	2020	2021	2022	2023	2024	2025
Rodents examined	10^3^ individuals	156.97	150.27	141.70	138.16	131.74	131.73
Ectoparasites examined	10^3^ specimens	1270.15	1235.89	1177.60	1456.70	1354.40	1507.08
Other materials examined	10^3^ samples	1.782	3.410	2.028	1.654	0.616	0.722
Bacteriological examinations	10^3^ samples	186.98	180.15	229.50	218.57	226.76	231.13
*Y. pestis* strains isolated	n	31	11	23	35	43	91
Isolation rate	per 10^5^ samples *	16.6	6.1	10.0	16.0	19.0	39.4
Real-time PCR tests	10^3^ samples *	1.20	1.62	9.75	11.96	13.28	53.42
Positive real-time PCR results	n	14	15	22	21	8	71
Real-time PCR positivity rate	per 10^3^ tests *	11.7	9.3	2.3	1.8	0.6	1.3
Biological assays	10^3^ samples	41.226	38.759	33.228	16.584	12.521	12.679
Total serological tests	10^3^ tests	160.45	156.52	135.32	138.26	155.42	137.38
Positive serological results	n	240	185	51	66	134	212

* Isolation rate was calculated as the number of *Y. pestis* strains isolated per 10^5^ bacteriologically examined samples. Real-time PCR positivity was calculated as the number of positive results per 10^3^ tests performed.

**Table 4:** Results of phenotypic characterization of 1526 *Y. pestis* strains and genotypic characterization of a representative subset of 75 whole-genome sequenced isolates.

Autonomous Plague Focus	Proportion of Strains Studied, %	Phenotypic Characteristics, %		Genotypic Characteristics, %	
		Typical	Atypical	Typical	Atypical
Balkhash	23.4	87.1	12.9	94.4	5.6
Moyynkum	15.6	97.3	2.7	98.3	1.7
Ili intermontane	17.0	97.3	2.7	99.2	0.8
Aral–Karakum	7.6	98.3	1.7	99.1	0.9
Taukum	7.4	97.3	2.7	100	–
North Aral	9.4	98.6	1.4	100	–
Ustyurt	5.2	98.7	1.3	100	–
Kyzylkum	4.4	100	–	100	–
Betpakdala	4.2	100	–	100	–
Arys-Kum-Daryalyktakyr	2.0	100	–	100	–
Pre-Ustyurt	1.3	100	–	100	–
Ural–Emba	0.5	100	–	100	–
Mangystau	0.4	100	–	100	–
Pre-Ustyurt (secondary)	0.3	100	–	100	–
Volga–Ural (sandy)	0.3	100	–	100	–
Ustyurt (secondary)	0.3	100	–	100	–
Mangyshlak	0.2	100	–	100	–
Volga–Ural (steppe)	0.1	100	–	100	–
Ural–Uil	0.1	100	–	100	–
Pre-Alakol	0.1	100	–	100	–
Saryjaz	0.1	100	–	100	–
Talas	0.1	100	–	100	–
Total (%)	100	94.9	5.1	97.5	2.5

## Data Availability

The data presented in this study are available from the corresponding author upon reasonable request. The data are not publicly available due to institutional and national restrictions related to epidemiological surveillance of particularly dangerous infections.

## References

[1] Perry R.D., Fetherston J.D. (1997). *Yersinia pestis*—Etiologic Agent of Plague. Clin. Microbiol. Rev..

[2] Gage K.L., Kosoy M.Y. (2005). Natural history of plague: Perspectives from more than a century of research. Annu. Rev. Entomol..

[3] World Health Organization (2021). Plague around the World, 2010–2020. Wkly Epidemiol. Rec..

[4] Centers for Disease Control and Prevention (CDC) About Plague: Maps and Statistics: Plague Worldwide. https://www.cdc.gov/plague/maps-statistics/index.html#cdc_data_surveillance_section_5-plague-worldwide.

[5] Popov N.V., Karnaukhov I.G., Kuznetsov A.A., Matrosov A.N., Ivanova A.V., Martsokha K.S., Kuklev E.V., Korzun V.M., Verzhutsky D.B., Chipanin E.V. (2024). Epidemiological situation on plague around the world and forecast of epizootic activity in the Russian Federation for 2024. Probl. Part. Danger. Infect..

[6] Popov N.V., Karnaukhov I.G., Matrosov A.M., Ivanova A.V., Kuznetsov A.A., Porshakov A.M., Pospelov M.V., Neishtadt Y.A., Korzun V.M., Verzhutsky D.B. (2026). Analysis of the global plague epidemiological situation in 2025 and forecast for 2026. Probl. Part. Danger. Infect..

[7] Atshabar B.B., Burdelov L.A., Izbanova U.A., Kozhakhmetova M.K., Aimakhanov B.K., Abdeliev Z.Z. (2015). Passport of regions of Kazakhstan for particularly dangerous infections. Quar. Zoonotic Infect. Kazakhstan.

[8] Abdel Z.Z., Erubaev T.K., Tokmurzieva G.Z., Aimakhanov B.K., Dalibaev Z.S., Musagalieva R.S., Zhumadilova Z.B., Meka-Mechenko V.G., Meka-Mechenko T.V., Matzhanova A.M. (2021). Demarcation of the boundaries of the Central Asian desert natural plague focus in Kazakhstan. Probl. Part. Danger. Infect..

[9] Aikimbaev A.M. (2006). Epidemic Potential of Natural Plague Foci of Kazakhstan.

[10] Abdel Z., Zhumadilova Z., Mussagalieva R., Tokmurzieva G., Yessimseit D., Abdeliyev B., Aimakhanov B., Meka-Mechenko V. (2025). Spatial and temporal characterization of *Yersinia pestis* in Kazakhstan. Environ. Anal. Health Toxicol..

[11] Popov N.V., Karnaukhov I.G., Kuznetsov A.A., Matrosov A.N., Ivanova A.V., Martsokha K.S., Korzun V.M., Verzhutsky D.B., Chipanin E.V., Kholin A.V. (2023). Improvement of Epidemiological Surveillance of Natural Plague Foci of the Russian Federation and the Forecast of Their Epizootic Activity for 2023. Probl. Part. Danger. Infect..

[12] Rivkus Y.Z., Blummer A.G. (2016). Plague Endemicity in the Deserts of Central Asia and Kazakhstan.

[13] Khamzin S.K. (1998). Plague Prevention in the Atyrau Region.

[14] Temiralieva G.A., Lukhnova L.Y., Arakelyan I.S., Martinevsky I.L. (1999). Anti-Plague Service of Kazakhstan: Historical Milestones.

[15] Popova A.Y., Kutyrev V.V. (2022). Atlas of Natural Plague Foci of Russia and Foreign Countries.

[16] World Health Organization Plague. https://www.who.int/news-room/fact-sheets/detail/plague.

[17] Rametov N., Abdel Z., Zhumadilova Z., Yessimseit D., Abdeliyev B., Mussagaliyeva R., Issaeva S., Althuwaynee O.F., Baygurin Z., Tabynov K. (2024). Historical assessment and mapping of human plague, Kazakhstan, 1926–2003. Emerg. Infect. Dis..

[18] Abdel Z., Zhumadilova Z., Mussagalieva R., Abdirassilova A., Rysbekova A., Issaeva S., Baitursyn B., Abdeliyev B., Otebay D., Jumagaziyeva A. (2025). Antibiotic Susceptibility Screening and Search for Resistance Genes in *Yersinia pestis* Clinical Isolates from Plague Outbreaks in Natural Foci of Kazakhstan (1926–2003). Microb. Drug Resist..

[19] Abdirassilova A.A., Yessimseit D.T., Kassenova A.K., Mussagalieva R.S., Tokmurzieva G.Z., Abdeliyev B.Z., Aimakhanov B.K. (2025). Whole genome sequencing of *Yersinia pestis*. PLoS Negl. Trop. Dis..

[20] Atshabar B., Nurtazhin S.T., Shevtsov A., Aimakhanov B., Abdeliev Z. (2021). Populations of *Rhombomys opimus* and vectors. Bull. Natl. Acad. Sci. Kazakhstan.

[21] Abdel Z., Zhumadylova Z., Aikimbayev A., Tokmurziyeva G., Mussagaliyeva R., Abdirassilova A., Issayeva S., Baitursyn B., Dalibayev Z., Abdeliyev B. (2025). Forecasting the spatial dynamics of the epizootic process plague among the wild animals in the desert plague foci of kazakhstan for the period 2020–2024. Based on gis technologies. Eurasian J. Appl. Biotechnol..

[22] Rametov N.M., Steiner M., Bizhanova N.A., Abdel Z.Z., Yessimseit D.T., Abdeliyev B.Z., Mussagalieva R.S. (2023). Mapping plague risk using models. GeoHealth.

[23] Atshabar B.B., Burdelov L.A., Sadovskaya V.P., Ageev V.S., Aubakirov S.A., Meka-Mechenko V.G., Meka-Mechenko T.V., Nekrasova L.E., Mussagalieva R.S., Sagiev Z.O. (2012). Atlas of Distribution of Particularly Dangerous Infections in Kazakhstan.

[24] Carlson C.J., Albery G.F., Merow C., Trisos C.H., Zipfel C.M., Eskew E.A., Olival K.J., Ross N., Bansal S. (2022). Climate change increases cross-species viral transmission risk. Nature.

[25] Ryan S.J., Carlson C.J., Mordecai E.A., Johnson L.R. (2019). Global expansion and redistribution of Aedes-borne virus transmission risk with climate change. PLoS Negl. Trop. Dis..

[26] Mamedzade F.U., Vanyan A.V., Avetisyan L.M., Torosyan L.S., Saakyan L.V., Yeritsyan S.B., Bekshin Z.M., Esmagambetova A.S., Zholshorinov A.Z., Zhumadilova Z.B. (2019). Methodological Recommendations: Epidemiological Surveillance in Natural Plague Foci of the CIS Countries.

[27] Stepanov V.M., Aubakirov S.A., Burdelov L.A., Burdelov A.S., Serzhanov O.S., Yakunin B.M., Pole S.B., Tleugabylova A.M., Fedorov Y.M., Rudenchik Y.V., Burdelov L.A. (1992). Guidelines for Plague Prevention in the Central Asian Desert Focus.

[28] World Health Organization (1999). Plague Manual: Epidemiology, Distribution, Surveillance and Control.

[29] Nekrasova L.E., Temiralieva G.A., Meka-Mechenko T.V., Zakaryan S.B., Stybaeva G.S. (2001). Guidelines for Study of Plague Strains.

[30] Meka-Mechenko T.V., Zakaryan S.B. (2011). Guidelines for Molecular Genetic Analysis of Plague Strains.

[31] Ditchburn J.-L., Hodgkins R. (2019). *Yersinia pestis*, a Problem of the Past and a Re-Emerging Threat. Biosaf. Health.

[32] Stenseth N.C., Samia N.I., Viljugrein H., Kausrud K.L., Begon M., Davis S., Leirs H., Dubyanskiy V.M., Esper J., Ageyev V.S. (2006). Plague dynamics are driven by climate variation. Proc. Natl. Acad. Sci. USA.

[33] Mills J.N., Gage K.L., Khan A.S. (2010). Potential influence of climate change on vector-borne and zoonotic diseases: A review and proposed research plan. Environ. Health Perspect..

[34] Sharan M., Vijay D., Yadav J.P., Bedi J.S., Dhaka P. (2023). Surveillance strategies for zoonotic diseases: A One Health approach. Sci. One Health.

[35] Ben-Ari T., Neerinckx S., Gage K.L., Kreppel K., Laudisoit A., Leirs H., Stenseth N.C. (2012). Plague and climate: Scales matter. PLoS Pathog..

[36] Reijniers J., Begon M., Ageyev V.S., Leirs H. (2014). Plague epizootic cycles in Central Asia. Biol. Lett..

[37] Achtman M., Morelli G., Zhu P., Wirth T., Diehl I., Kusecek B., Vogler A.J., Wagner D.M., Allender C.J., Easterday W.R. (2004). Microevolution and history of the plague bacillus, *Yersinia pestis*. Proc. Natl. Acad. Sci. USA.

[38] Cui Y., Yu C., Yan Y., Li D., Li Y., Jombart T., Weinert L.A., Wang Z., Guo Z., Xu L. (2013). Historical variations in mutation rate in an epidemic pathogen, *Yersinia pestis*. Proc. Natl. Acad. Sci. USA.

[39] Dentovskaya S.V., Kadnikova L.A., Kislichkina A.A., Bogun A.G., Anisimov A.P. (2018). Intraspecific diversity of *Yersinia pestis*. Russ. J. Infect. Immun..

[40] Eisen R.J., Enscore R.E., Biggerstaff B.J., Reynolds P.J., Ettestad P., Brown T., Pape J., Tanda D., Levy C.E., Engelthaler D.M. (2007). Human plague in the southwestern United States, 1957–2004: Spatial models of elevated risk of human exposure to *Yersinia pestis*. J. Med. Entomol..

[41] Stenseth N.C., Atshabar B.B., Begon M., Belmain S.R., Bertherat E., Carniel E., Gage K.L., Leirs H., Rahalison L. (2008). Plague: Past, present, and future. PLoS Med..

[42] Jones K.E., Patel N.G., Levy M.A., Storeygard A., Balk D., Gittleman J.L., Daszak P. (2008). Global trends in emerging infectious diseases. Nature.

[43] Gage K.L., Burkot T.R., Eisen R.J., Hayes E.B. (2008). Climate and vectorborne diseases. Am. J. Prev. Med..

